# K-12 students’ basic psychological needs satisfaction in online learning: perceived level, group difference, and its influence

**DOI:** 10.3389/fpsyg.2026.1561201

**Published:** 2026-03-02

**Authors:** Xuemei Bai, Rifa Guo

**Affiliations:** 1 School of Teacher Education, Ningxia University, Yinchuan, China; 2 Institute of Education, Tsinghua University, Beijing, China

**Keywords:** basic psychological needs, group difference, online learning, online learning behavioral engagement, online learning cognitive engagement, online learning emotional engagement

## Abstract

**Background:**

In the post-pandemic educational landscape, online learning has become increasingly embedded in K-12 education. Self-determination theory claims that satisfying basic psychological needs is critical in student learning. Despite numerous empirical researches on basic psychological needs, there remains limited understanding of K-12 students’ perceived level, group differences of basic psychological needs satisfaction in online learning and its influence on online learning engagement.

**Objectives:**

This study aims to (i) examine K-12 students’ perceived levels of basic psychological needs satisfaction in online learning; (ii) analyze group differences based on gender and grade level; and (iii) test the influence of need satisfaction on cognitive, behavioral, and emotional engagement in online learning.

**Methodology:**

A quantitative study was employed. Data were collected from 1,073 K-12 students across 10 schools in China using stratified random sampling. Data were analyzed using descriptive statistics, independent samples *t*-tests, one-way ANOVA, Pearson correlations, and multiple regression analyses.

**Results:**

Results indicated that most students reported moderate levels of psychological needs satisfaction, with few reporting high levels. Significant differences were found across grade levels: middle school students reported higher satisfaction than both elementary and high school students, and elementary students scored higher than high school students; no significant gender differences were observed. Regression analyses revealed that autonomy and competence significantly predicted all three dimensions of engagement. Competence emerged as the strongest predictor. Relatedness significantly predicted emotional and behavioral engagement but not cognitive engagement.

**Conclusion and implications:**

These findings extend Self-Determination Theory to the context of K-12 online learning and highlight the differential roles of the three basic needs. The results underscore the importance of designing online instruction that enhances competence and autonomy to promote student engagement. Educational policymakers and practitioners should consider grade-specific strategies to better support students’ basic psychological needs in digital environments.

## 1 Introduction

The outbreak of COVID-19 has led to unprecedented growth in online learning, even though the epidemic has been effectively controlled and offline teaching has resumed. As an important form of learning, research on online learning is still ongoing (Mendoza et al., 2023). Guided by Self-Determination Theory (SDT) (Ryan and Deci, 2020)—a macro-theory of human motivation that posits autonomy, competence, and relatedness as three universal psychological needs essential for intrinsic motivation and well-being—this study examines how these needs operate in the context of K–12 online learning. SDT proposes that all individuals possess three universal psychological needs: autonomy, competence, and relatedness. As a macro-theory of human motivation, SDT posits that these needs function as essential nutrients for intrinsic motivation and psychological wellbeing, and their satisfaction—or frustration—mediates the impact of environmental supports on behavioral outcomes (Ryan and Deci, 2020). Within the SDT framework, autonomy refers to the experience of volition and self-endorsed choice in one’s actions, rather than compliance with external pressures; competence denotes the sense of effectiveness in mastering tasks and achieving desired outcomes through one’s skills; and relatedness reflects the feeling of meaningful connection, care, and belonging with others in the learning environment (Deci and Ryan, 2000; Ryan and Deci, 2020).

SDT has been widely used in face-to-face teaching (Niemiec and Ryan, 2009; Wang et al., 2019); a large number of studies found that students’ perception of basic psychological needs satisfaction can predict learning performance, learning persistence, course satisfaction (Ryan and Deci, 2020). However, the extension of SDT to online learning remains nascent. The contribution of SDT to online learning has not received much attention, and few studies have paid attention to students’ basic psychological needs satisfaction in online learning (Hsu et al., 2019). After a systematic analysis of relevant studies, Ryan and Deci (2020) call for future research on the application of SDT in e-learning and distance learning. Recently, Hsu et al. (2019) and Luo et al. (2021) provide empirical evidence for the applicability of SDT to online learning. Moreover, students’ perception of basic psychological needs satisfaction in online learning has attracted the attention of researchers. Fabriz et al. (2021) found that, compared with asynchronous online learning, students who participated in synchronous online learning had a higher perception satisfaction of competence and relatedness. Wong (2022) explored middle school students’ perception of basic psychological needs satisfaction in blended learning and found that they can perceive a certain level of autonomy needs, competence needs, and relatedness.

Basic psychological need support is critical given the diversity of learners (Ryan and Deci, 2020). SDT acknowledges the differences between people. It is important to appreciate individual differences in how basic needs are fulfilled (Ryan and Deci, 2020). The means through which needs are satisfied (versus thwarted) vary as a function of age, gender, and culture. Thus, the same behavior can be satisfying for one group and thwarting for another (Ryan and Deci, 2002). Notably, developmental research within the SDT framework indicates that early and mid-adolescence (i.e., junior high school years) represent a peak period for autonomy-seeking and heightened vulnerability to controlling environments (Soenens and Vansteenkiste, 2005). In contrast, older adolescents in senior high school often face increased academic demands and future-oriented pressures, which may shift their motivational dynamics and alter their sensitivity to basic psychological need support. Therefore, a theoretically grounded examination of how grade levelnamics and alter their sensitivity s and futurelearning had a higher perception lacking in the online learning literature. Therefore, studies examining whether students of different characteristics have different perceptions of these three needs has important practical significance for improving different students’ basic psychological needs satisfaction. Researchers should consider the possible effects of demographic variables such as participants’ gender, age, and grade (Turk et al., 2022). However, interventions focused on satisfying students’ basic psychological needs in online learning remain in the early stages (Mendoza et al., 2023), as far as we know, no studies have focused on the differences in the perception of basic psychological needs of students of different genders and grades, in online learning settings.

Learning engagement is important for online learning outcomes (Huang and Wang, 2023). Theoretically, learning engagement is often seen as an outcome of motivation. The core hypotheses of SDT in education is that more autonomous forms of motivation will enhance students’ engagement. Basic psychological need support from teachers and parents facilitates such motivation, whereas need thwarting undermines it (Ryan and Deci, 2020). As a well-known motivation theory, SDT claims that fulfilling the three basic psychological needs in an energy source activates students to be engaged in learning. These hypotheses have been well supported across hundreds of studies in the traditional face-to-face teaching context (e.g., Kurt and Tas, 2018). Recently, researchers have attempted to examine the contribution of basic psychological needs satisfaction to online learning engagement. Chiu (2022) found that students perceived satisfaction with the three basic psychological needs significantly positively affected their online learning behavioral, cognitive, and emotional engagement. However, Huang and Wang (2023) found a significant negative correlation between relatedness satisfaction and cognitive engagement in online learning. This inconsistency underscores the need for further empirical validation—particularly across diverse age groups and cultural contexts—to clarify the directional and differential roles of each need in shaping multidimensional engagement. These conclusions are inconsistent or oppose one another; thus, further verification is needed to confirm the contribution of the three basic psychological needs to student online learning engagement.

Therefore, grounded in SDT’s proposition that basic psychological need satisfaction serves as the proximal antecedent of engagement and is moderated by developmental and contextual factors, one of the purposes of the present study is to explore K-12 students including primary, junior middle, and senior high school students in the Chinese educational system, their perception level and group differences in basic psychological needs satisfaction in online learning. Meanwhile, we also aim to investigate the influence of basic psychological needs satisfaction on online learning engagement. Accordingly, the research questions are as follows:

*RQ1*: What is K-12 students’ overall perception level of basic psychological needs satisfaction in online learning?

*RQ2*: Are there differences in the perception of basic psychological needs satisfaction among students of different genders and grades?

*RQ3*: Does students’ perception of the three basic psychological needs satisfaction significantly influence their online learning engagement? If so, is there a difference?

## 2 Literature review

### 2.1 SDT and basic psychological needs

SDT assumes people are inherently prone to learning, mastery, and connection with others. However, these tendencies are not seen as automatic—they require supportive conditions. Accordingly, SDT primarily focuses on how much educational intervention meets or frustrates these basic needs. Satisfaction with basic psychological needs has significantly influenced students’ learning outcomes in face-to-face teaching. Previous studies show that SDT can predict learning outcomes (Fabriz et al., 2021). Students with more autonomy support from teachers have better grades (Vallerand et al., 1997), greater internalization for learning activities, and lower dropout (Hardré and Reeve, 2003). Furthermore, students’ perception of basic psychological needs satisfaction can predict students’ learning performance, learning persistence, course satisfaction (Ryan and Deci, 2020). Furthermore, Fabriz et al. (2021) revealed a more positive learning experience associated with greater fulfillment of students’ basic psychological needs.

### 2.2 Basic psychological needs in online learning settings

Initially, Chen and Jang (2010) proposed a model for online learner motivation based on SDT. With the continuous expansion of online education practice, more researchers are paying attention to SDT in online learning. With the widespread popularity of online learning in recent years, given the importance of meeting students’ basic psychological needs for online learning, in addition to focusing on the application of SDT (Hsu et al., 2019; Luo et al., 2021; Wong, 2022; Bai and Gu, 2024) and students’ perception of basic psychological needs satisfaction in online learning (Fabriz et al., 2021; Wong, 2022), researchers focus on how to satisfy students’ basic psychological needs in online learning. Alamri et al. (2020) examined the perceived efficacy of using personalized learning activities to support students’ psychological need satisfaction in an online course. Participants felt the learning pathways were helpful in their learning, but, unfortunately, which created a feeling of a lack of connectedness, did not support students’ feelings of relatedness. Ismailov and Chiu (2022) tested whether the universal design for learning can support all students’ basic psychological needs satisfaction and found that, although the universal design supported autonomy and competence, it nonetheless failed to satisfy learners’ relatedness fully. However, investigating students’ perceived level of basic psychological needs satisfaction and group differences can bring valuable evidence to design targeted interventions for different groups and promote their perceived satisfaction with basic psychological needs, which has not been extensively tested.

### 2.3 Basic psychological needs and online learning engagement

Given the separation between teachers and students in online learning, the importance of online learning engagement is self-evident. In general, engagement comprises three dimensions: behavioral, cognitive, and emotional (Fredricks et al., 2004). Behavioral engagement is the basic form of participation in learning, which is explicit and observable. It mainly includes the concrete behavior of students in the learning process in terms of attention, participation, effort, intensity, or persistence. Cognitive engagement refers to students’ psychological involvement in learning activities through metacognitive strategies, such as goal setting, self-regulation, and self-monitoring, as well as their intellectual efforts to master the learning content. It emphasizes that students use sophisticated rather than superficial learning strategies to master and control psychological efforts in the learning process. Emotional engagement refers to students’ emotional responses to teachers, peers, learning activities, school experience, and emotions that promote task completion (Fredricks et al., 2004; Sinatra et al., 2015).

Researchers have focused on the influence of students’ perceived basic psychological needs satisfaction on online learning motivation and engagement. Mendoza et al. (2023) tested the effect of need-supportive task instruction on students’ intrinsic motivation in an online language learning task and revealed that the need-supportive task instructions had a medium effect size on intrinsic motivation. Luo et al. (2021) revealed that the three basic needs are associated with intrinsic motivation. Chiu (2022) investigated how the three perceived psychological needs affected student engagement in online learning and found that all of the needs were predictors engagement; perceived relatedness is the most important predictor of behavioral, emotional, and agentic engagement and has the largest effect on these three engagements. However, Huang and Wang (2023) revealed positive significant correlations between relatedness and behavioral and emotional engagement but a negative significant correlation between relatedness and cognitive engagement. The current findings about the influence of the three basic psychological needs on different dimensions of online learning engagement are inconsistent, even opposite. Therefore, this study aims to investigate further the effects of the three basic psychological needs on online learning engagement dimensions and their differences.

## 3 Materials and methods

### 3.1 Research instruments

The measurement of students’ basic psychological needs drew on the Basic Psychological Needs Scale for Online Learning developed by Rösler et al. (2016), which has demonstrated strong reliability and validity in online learning contexts (e.g., Fabriz et al., 2021). To enhance developmental appropriateness for younger K–12 learners and address potential gaps in relatedness assessment, we incorporated four additional items from Wong (2022), whose scale was validated specifically in Chinese blended learning environments. The final scale comprised 13 items: 3 assessing autonomy, 6 for competence, and 4 for relatedness. Online learning engagement was measured using Elmaadaway’s (2018) 17-item Online Student Engagement scale, which is grounded in Fredricks et al.’s (2004) tripartite framework. The scale includes five items for emotional engagement, six for cognitive engagement, and six for behavioral engagement. All scales employed a five-point Likert format.

The original scales were developed and validated in English. We conducted a rigorous forward-backward translation process involving two bilingual researchers and one educational psychologist to adapt them into Chinese. The translated versions were pilot-tested with 30 students to ensure clarity and cultural relevance.

### 3.2 Data collection

The data were collected in October 2022 during a period of widespread COVID-19 outbreaks in China, when schools shifted to full-time online instruction and students learned from home. Online learning was delivered primarily through synchronous video conferencing (e.g., DingTalk, Tencent Meeting) and supplemented with asynchronous activities (e.g., recorded lectures, online assignments via WeChat). Instruction was organized and delivered by teachers as part of the official curriculum, ensuring that students’ experiences reflected structured, school-led online learning rather than informal or supplemental education.

To enhance representativeness within the regional context, we employed a two-stage stratified random sampling design. First, one western province was selected purposively due to its socio-economic diversity and typical challenges in digital infrastructure—making it a critical site for studying equitable online learning. Within this province, we stratified by administrative level: provincial capitals, prefecture-level cities, county towns, and rural townships/villages. From each stratum, public schools were randomly selected from the provincial education bureau’s registry using a computer-generated random number sequence. In total, 10 schools were selected (3 from provincial capitals, 2 from prefecture-level cities, 3 from county towns, and 2 from rural areas).

Second, within each selected school, we identified all eligible grades (Grades 4–12) and randomly selected 2–6 intact classes per school using class rosters provided by school administrators. This approach ensured inclusion across developmental stages while maintaining feasibility. An electronic questionnaire was created on Wenjuanxing (a secure Chinese survey platform). For students in Grades 4–6, a short audio-guided instruction explained key terms (e.g., “autonomy,” “engagement”) before the survey began to ensure age-appropriate comprehension. Participation was voluntary and anonymous; no personal identifiers were collected. Parents received an information sheet and could opt their child out; students provided active assent before proceeding.

Overall, 1155 Chinese K-12 students from Mainland China participated in the survey, with the majority being of Han ethnicity and native Mandarin speakers. All the collected data were preprocessed to ensure no missing or abnormal values appeared in the overall sample data. We omitted 82 samples in the preprocessing, leaving 1,073 samples as valid sample data. Given that the multiple regression model included three predictors, the sample size of *N* = 1,073 exceeds the commonly recommended minimum of 10–15 participants per predictor (Green, 1991), ensuring adequate statistical power. Among them, 526 (49%) were female and 547(51%) were male. A total of 341(32%) participants were elementary school students (Grades 4–6), 346(32%) were middle school students (Grades7–9), and 386(36%) were high school students (Grades 10–12), with ages ranging from 10 to 18 years. The sample was drawn from four types of regions: provincial capitals (*n* = 280, 26.1%), prefecture-level cities (*n* = 180, 16.8%), county towns (*n* = 380, 35.4%), and rural towns or villages (*n* = 233, 21.7%). All participants were enrolled in public schools.

### 3.3 Reliability and validity analysis

Reliability was assessed using SPSS 17.0. Cronbach’s α coefficients for the basic psychological needs and online learning engagement scales were 0.965 and 0.940, respectively, indicating high internal consistency. Specifically, α coefficient of autonomy needs, competence needs, and relatedness were 0.892, 0.954, and 0.917, respectively. α coefficients of online learning emotional engagement, behavioral engagement, and cognitive engagement were 0.828, 0.940, and 0.927, respectively.

Exploratory factor analysis (EFA) supported structural validity. For the basic psychological needs scale, KMO = 0.958, Bartlett’s test: χ^2^ (1,186) = 14,547.999, *p* < 0.001, indicating sampling adequacy and significant sphericity. A three-factor solution accounted for 82.20% of the total variance n atonomy (33.11%), competence (25.21%), and relatedness (23.87%), and relatedness (23 > 1. For the online learning engagement scale, KMO = 0.939, χ^2^ (1,186) = 16,721.377, *p* < 0.001. The three-factor structure explained 73.92% of the variance: emotional engagement (28.72%), cognitive engagement (27.59%), and behavioral engagement (17.61%), with eigenvalues exceeding 1, confirming good construct validity.

## 4 Results

### 4.1 Students’ overall perception level of basic psychological needs

Descriptive statistical analysis was run to answer research question 1. Students’ perception of basic psychological needs satisfaction levels are summarized in Table 1.

**TABLE 1:** Descriptive statistics of K-12 students’ perceptions of basic psychological needs (*N* = 1,073).

Dimensions	M	SD	Min	Max	Level (f/%)					
					Low		Medium		High	
Autonomy needs	12.17	2.63	3.00	15.00	230	21.4	493	45.9	350	32.6
Competence needs	23.16	5.55	6.00	30.00	102	9.5	685	63.8	286	26.7
Relatedness	15.69	3.70	4.00	20.00	94	8.8	694	64.7	285	26.6
Basic psychological needs	51.02	11.05	13.00	65.00	217	20.2	600	55.9	256	23.9

Table 1 shows that students’ basic psychological needs satisfaction scores range from 13 to 65, and the average score is 51.02 (SD = 11.05). Moreover, 23.9% of the students’ perception levels are high, 55.9% of the students’ perception levels are medium, and 20.2% of the students’ perception levels are low. More than half of the students (55.9%) reported a medium level of basic psychological needs satisfaction, while 23.9% reported a high level. Furthermore, students’ perception level of competence needs is the highest (23.16), followed by relatedness (15.69), and their perception level of autonomy needs is the lowest (12.17). In online learning, the largest proportion of students reported a medium level of autonomy (45.9%), competence (63.8%), and relatedness (64.7%).

### 4.2 Group differences of basic psychological needs

Independent sample *t*-test and one-way ANOVA were run to answer research question 2. First, an independent sample *t*-test was conducted to analyze the differences of students of different genders in their perception of basic psychological needs satisfaction and its three sub-dimensions. Levene’s test confirmed homogeneity of variances for all variables (all *p* > 0.05). The analysis results are shown in Table 2. The results indicated no statistically significant difference (*p* > 0.05) of students of different genders in the perceived level of basic psychological needs satisfaction and its three dimensions (all *p* > 0.05; Cohen’s d ranging from 0.02 to 0.07, indicating negligible effect sizes).

**TABLE 2:** Group differences among students of different genders (*n* = 1,073).

Psychological need dimension	Gender	*M*	SD	*t*	*p*
Autonomy needs	Male	12.14	2.80	−0.366	0.714
	Female	12.20	2.45		
Competence needs	Male	23.35	5.67	1.148	0.251
	Female	22.96	5.42		
Relatedness	Male	15.76	3.82	0.623	0.534
	Female	15.62	3.57		
Basic psychological needs	Male	51.26	11.48	0.697	0.486
	Female	50.79	10.61		

Second, a one-way ANOVA was run, and Levene’s test indicated homogeneity of variance (all *p* > 0.05). Post hoc comparisons were performed using the Bonferroni correction to control for multiple comparisons. The results (Table 3) showed significant differences among grade levels in autonomy needs [*F*(2, 1,070) = 31.46, *p* < 0.001, η^2^ = 0.055], competence needs (*F* = 34.29, *p* < 0.001, η^2^ = 0.060), relatedness (*F* = 25.99, *p* < 0.001, η^2^ = 0.046), and overall basic psychological needs satisfaction (*F* = 34.96, *p* < 0.001, η^2^ = 0.061). Middle school students scored significantly higher than both elementary and high school students on all dimensions. Elementary school students also scored higher than high school students on autonomy, competence, and total needs, but not on relatedness.

**TABLE 3:** Group differences of the perception of basic psychological needs among different grade (*n* = 1,073).

Psychological need dimension	Grade	*M*	SD.	*F*	Bonferroni *post-hoc* comparisons
Autonomy needs	Elementary school student	12.38	2.70	31.457**	Middle school > Elementary school Middle school > High school Elementary school > High school
	Middle school student	12.84	2.36		
	High school student	11.38	2.60		
Competence needs	Elementary school student	23.52	5.40	34.289**	Middle school > Elementary school Middle school > High school Elementary school > High school
	Middle school student	24.71	5.14		
	High school student	21.46	5.57		
Relatedness	Elementary school student	15.50	3.85	25.985**	Middle school > Elementary school Middle school > High school
	Middle school student	16.79	3.46		
	High school student	14.89	3.53		
Basic psychological needs	Elementary school student	51.40	11.04	34.958**	Middle school > Elementary school Middle school > High school Elementary school > High school
	Middle school student	54.34	10.06		
	High school student	47.73	11.02		

**P < 0.05.

### 4.3 The associations between students’ basic psychological needs and online learning engagement

Correlation analysis and multiple regression analysis were conducted to address research question 3. First, Pearson’s correlation coefficient was computed using SPSS21.0 to assess the relationships among the variables. The results show that students’ perception of autonomy needs, competence needs, relatedness, online learning emotional engagement, cognitive engagement, and behavioral engagement are all significantly correlated (Table 4). All correlations were significant at *p* < .01, and the majority exceeded 0.50, which may be considered large in magnitude according to conventional benchmarks (Cohen, 1988), though interpretation should consider the context of educational research. As shown in Table 4, all three basic psychological needs were strongly positively correlated with each other (*r*s ranging from 0.738 to 0.809), explaining between 54.5 and 65.4% of shared variance. All needs were moderately to strongly associated with the three types of engagement, with the strongest associations observed between competence needs and behavioral engagement (*r* = 0.702, *r*^2^ = 49.3%) and between cognitive engagement and behavioral engagement (*r* = 0.764, *r*^2^ = 58.4%). These bivariate associations support the hypothesized statistical links between needs satisfaction and online learning engagement.

**TABLE 4:** Pearson correlation matrix with effect size interpretations.

Variable	1	2	3	4	5	6
Autonomy needs	–					–
Competence needs	0.801** (64.2%)					
Relatedness	0.738** (l54.5%)	0.809** (65.4%)				
Emotional engagement	0.492** (24.2%)	0.525** (27.6%)	0.510** (26.0%)			
Cognitive engagement	0.587** (34.5%)	0.600** (36.0%)	0.522** (27.2%)	0.509** (25.9%)		
Behavioral engagement	0.688** (47.3%)	0.702** (49.3%)	0.638** (40.7%)	0.528** (27.9%)	0.764** (58.4%)	

**P < 0.05 Effect sizes. 0.10 = small, 0.30 = medium, 0.50 = large (Cohen, 1988). Values below the diagonal include effect size interpretation (e.g., “large”) and shared variance (r^2^, expressed as percentage) in parentheses.

**FIGURE 1:**
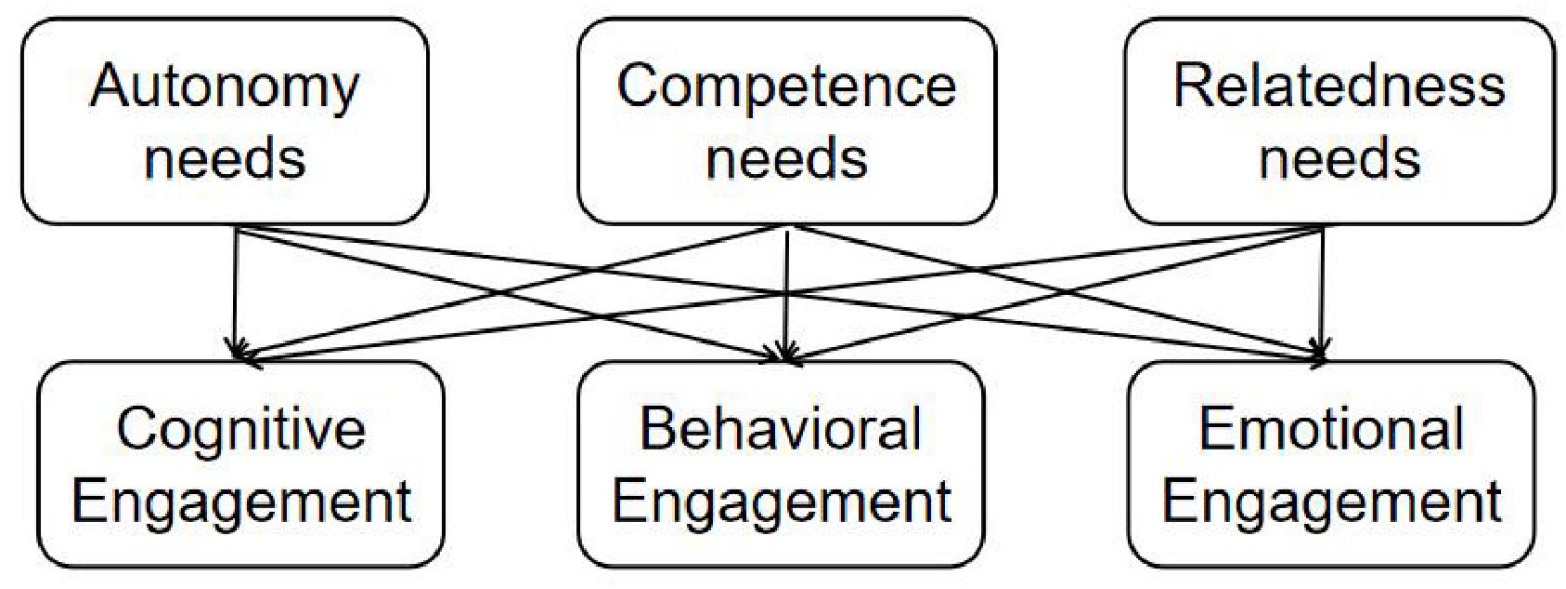
Path diagram of the regression framework showing the hypothesized associations between basic psychological needs and online learning engagement. The arrows indicate predictor–outcome relationships examined using multiple regression analysis.

To clarify the hypothesized relationships tested in the multiple regression analyses, Figure 1 presents a path diagram of the regression framework. The model illustrates how the three basic psychological needs, autonomy, competence, and relatedness, are examined as concurrent correlates of the three dimensions of online learning engagement: cognitive, behavioral, and emotional engagement.

First, regarding regression analysis, multicollinearity diagnostics were conducted for all regression models. The variance inflation factor (VIF) values for autonomy needs (VIF = 2.986), competence needs (VIF = 3.938), and relatedness (VIF = 3.094) were consistent across models and well below the threshold of 5.0. Tolerance values ranged from 0.254 to 0.335, exceeding the 0.10 criterion. These results indicate that multicollinearity does not pose a concern in any of the regression analyses. In addition, assumption checks were conducted separately for each regression model. Linearity was supported in all models. Homoscedasticity was generally met, although Model 3 (predicting behavioral engagement from the three basic psychological needs) showed mild heteroscedasticity, which was addressed using robust standard errors.

**FIGURE 2:**
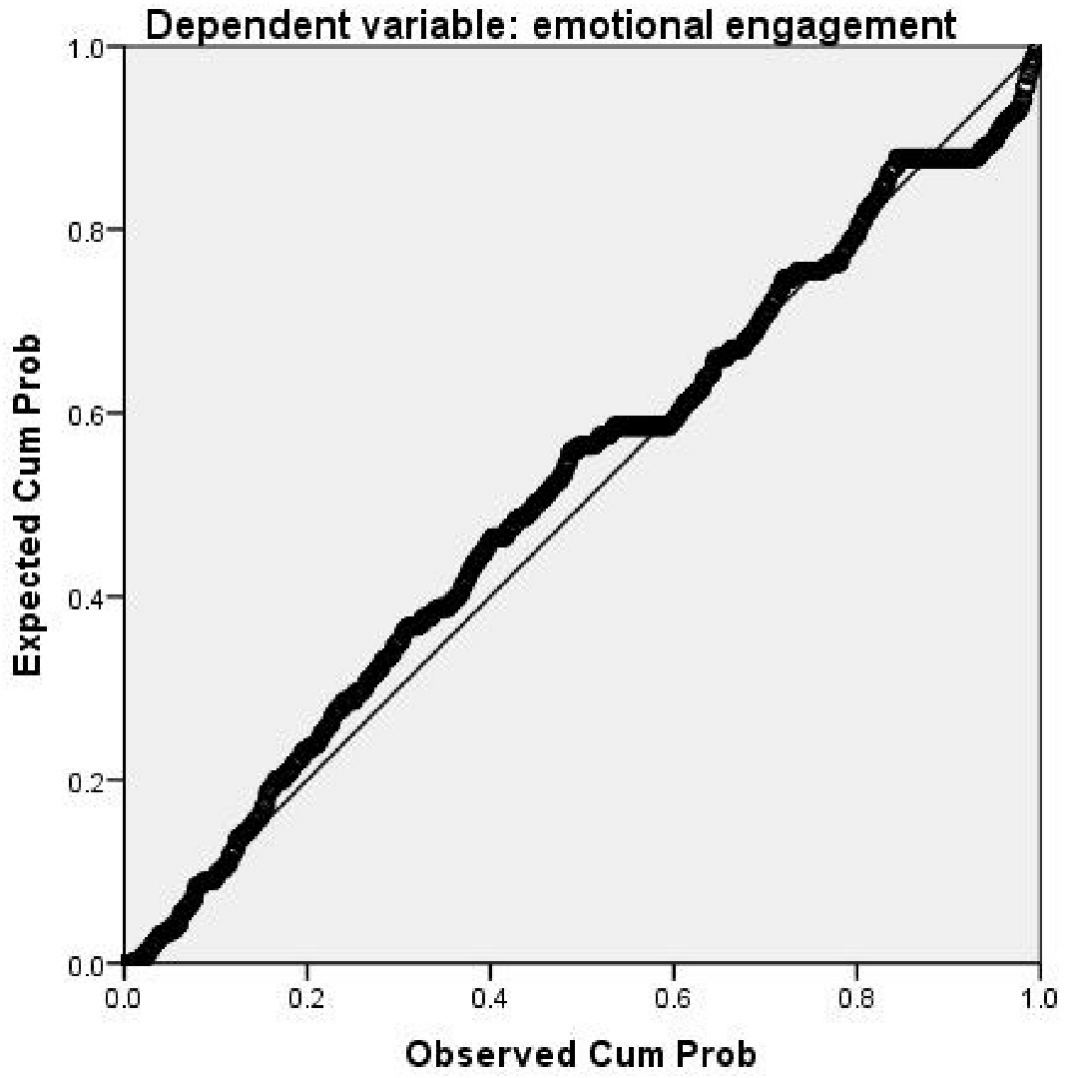
Normal probability plot of standardized regression residuals for emotional engagement, showing approximate normality and thus supporting the assumption of normally distributed errors in the linear model.

The results (Table 5) of the multiple linear regression analysis examining the association between the three basic psychological needs dimensions and online learning emotional engagement show that autonomy needs, competence needs, and relatedness accounted for 30.2% (adjusted *R*^2^ = 0.302) of the variability in online learning emotional engagement; the Durbin Watson value is 2.053. The overall regression models was statistically significant (*F* = 155.439, *p* < 0.001). Specifically, autonomy needs (β = 0.149, *P* < 0.001), competence needs (β = 0.236, *P* < 0.001), and relatedness (β = 0.208, *P* < 0.001) were each significantly associated with students’ online learning emotional engagement. The P-P plots showed no evidence of violations of normality and linearity, as seen in Figure 2.

**TABLE 5:** Regression analysis of basic psychological needs on different dimensions of online learning engagement (*N* = 1,073).

Model	*B*	SE	Beta	*t*	*p*	95.0% confidence interval for B	*R* ^2^
Autonomy needs— > EE	0.271	0.080	0.149	3.387	0.001	[0.114,0.428]	0.302
Competence needs— > EE	0.203	0.044	0.236	4.667	0.000	[0.118,0.289]	
Relatedness— > EE	0.269	0.058	0.208	4.641	0.000	[0.155,0.383]	
Autonomy needs— > CE	0.580	0.083	0.288	6.995	0.000	[0.418,0.743]	0.390
Competence needs— > CE	0.327	0.045	0.343	7.244	0.000	[0.239,0.416]	
Relatedness— > CE	0.046	0.060	0.032	0.763	0.445	[-0.072,0.164]	
Autonomy needs— > BE	0.658	0.073	0.322	8.987	0.000	[0.514,0.801]	0.540
Competence needs— > BE	0.337	0.040	0.347	8.449	0.000	[0.259,0.415]	
Relatedness— > BE	0.174	0.053	0.120	3.280	0.001	[0.070,0.278]	

EE, CE, and BE refer to emotional engagement, cognitive engagement, and behavioral engagement, respectively.

**FIGURE 3:**
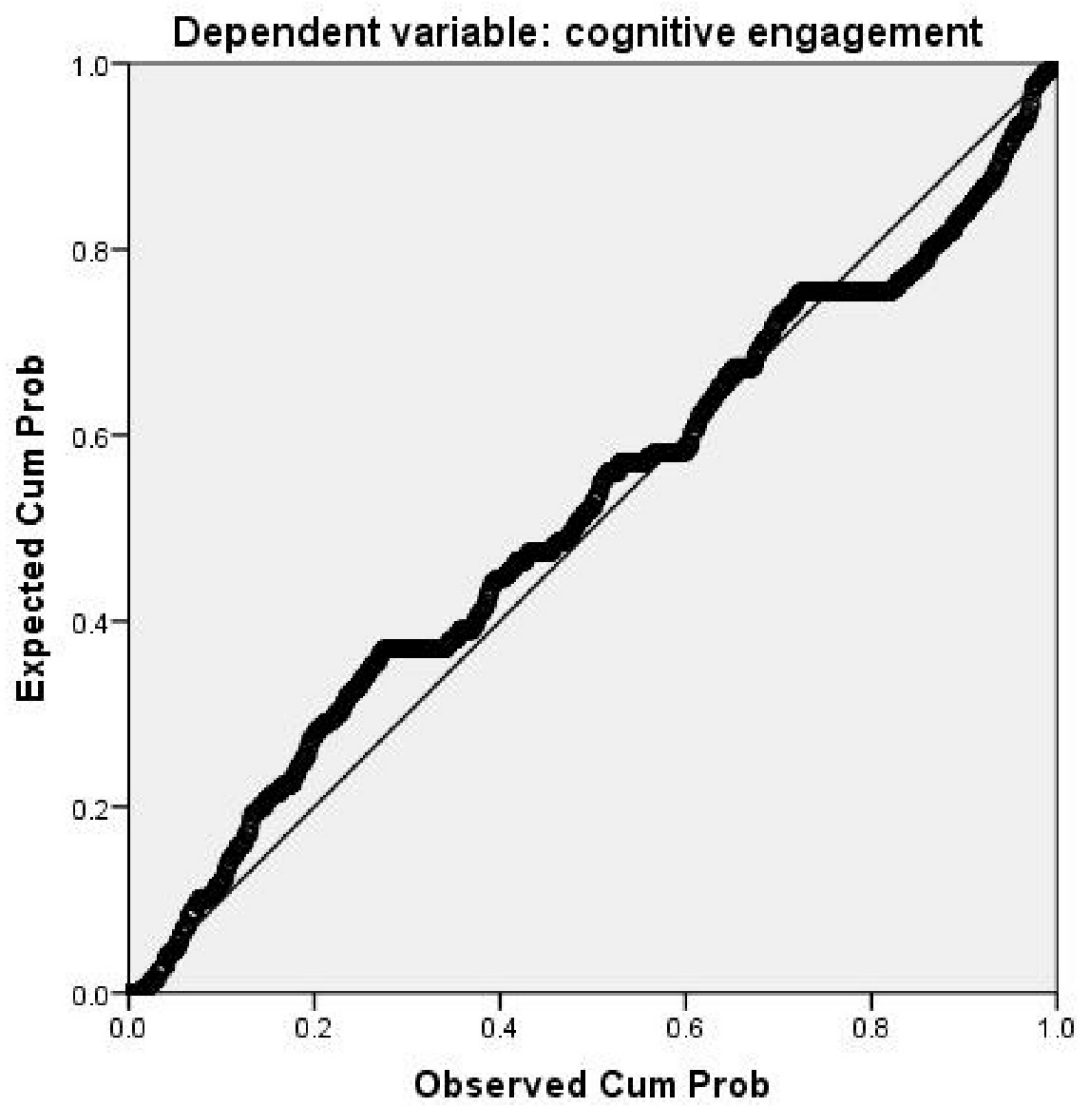
Normal probability plot of standardized regression residuals for cognitive engagement, showing approximate normality and thus supporting the assumption of normally distributed errors in the linear model.

The results (Table 5) of multiple linear regression analysis of the association between the three basic psychological needs and online learning cognitive engagement show that autonomy needs, competence needs, and relatedness accounted for 39.0% (adjusted *R*^2^ = 0.390) of the variability in online learning cognitive engagement; the Durbin Watson value is 1.793. The overall model was statistically significant (*F* = 229.413, *p* < 0.001). Specifically, autonomy needs (β = 0.288, *P* < 0.001) and competence needs (β = 0.343, *P* < 0.001) were significantly associated with online learning cognitive engagement, whereas relatedness (β = 0.032, *P* = 0.445) was not significantly associated with online learning cognitive engagement. The P-P plots showed no evidence of violations of normality and linearity, as seen in Figure 3.

**FIGURE 4:**
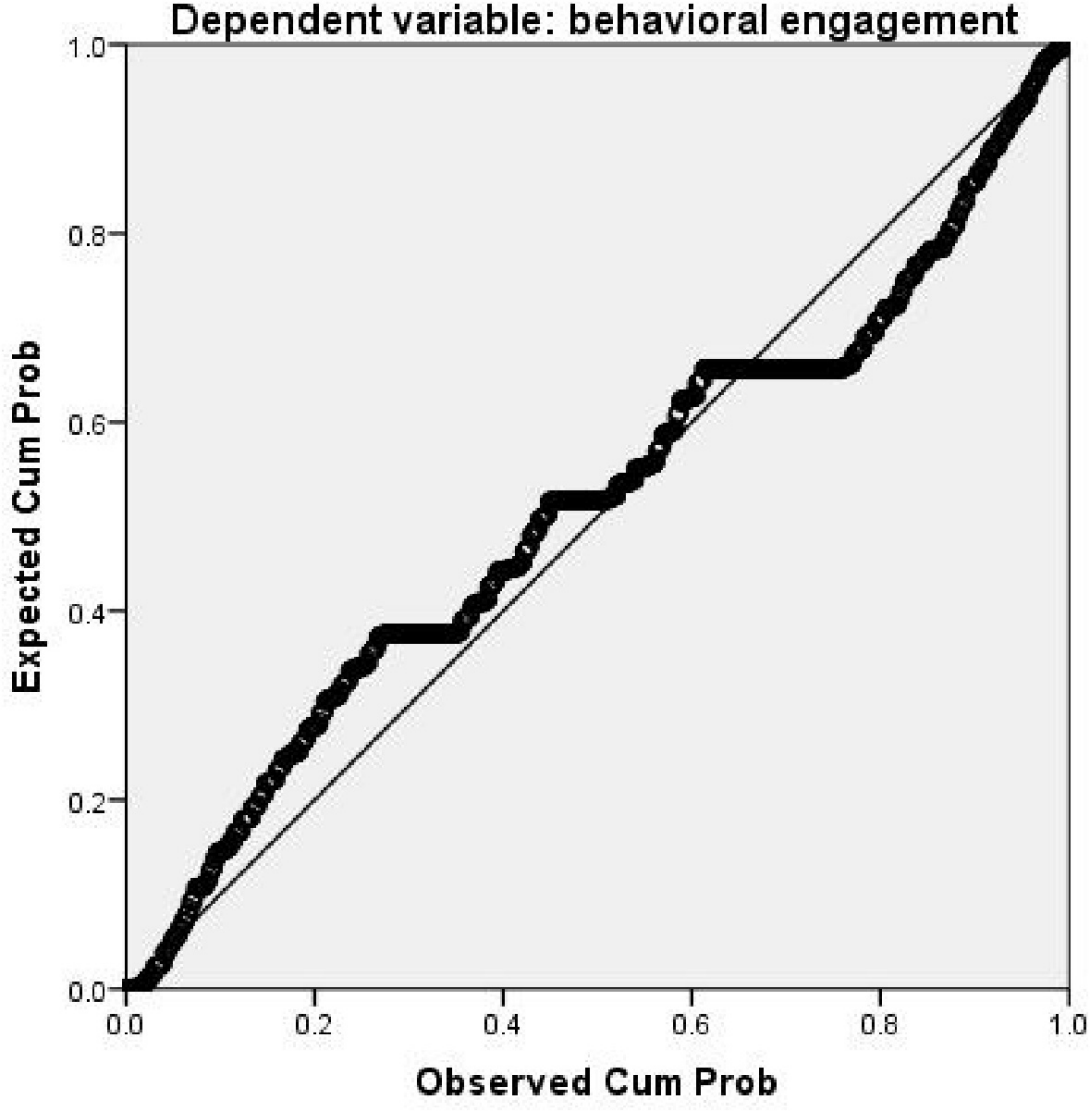
Normal probability plot of standardized regression residuals for behavioral engagement, showing approximate normality and thus supporting the assumption of normally distributed errors in the linear model.

The results (Table 5) of multiple linear regression analysis of the association between the three basic psychological needs and online learning behavioral engagement show that autonomy needs, competence needs, and relatedness accounted for 54.0% (adjusted *R*^2^ = 0.540) of the variability in online learning behavioral engagement; the Durbin Watson value is 1.969. The overall model was statistically significant (*F* = 420.509, *p* < 0.001). Specifically, autonomy needs (β = 0.322, *P* < 0.001), competence needs (β = 0.347, *P* < 0.001), and relatedness (β = 0.120, *P* < 0.001) were significantly associated with online learning behavioral engagement. The P-P plots showed no evidence of violations of normality and linearity, as seen in Figure 4.

In terms of relative strength of association, competence needs (β = 0.236) have the greatest influence on online learning emotional engagement, followed by relatedness (β = 0.208) and then autonomy needs (β = 0.149). Competence needs (β = 0.343) have a greater influence on online learning cognitive engagement than autonomy needs (β = 0.288). Competence needs (β = 0.347) also have the greatest influence on online learning behavioral engagement, followed by autonomy needs (β = 0.322) and relatedness (β = 0.120). Therefore, competence needs greatly influence all three online learning engagement dimensions. These findings suggest that competence needs are consistently and most strongly associated with all three dimensions of online learning engagement in this sample.

## 5 Discussion

### 5.1 Discussion for RQ1: perception levels

The present study found that more than half (55.9%) of the students’ perception levels of basic psychological needs are medium, 20.2% of the students’ perception levels are low, and only 23.9% of the students’ perception levels are high. Students’ perception levels of basic psychological needs depend on SDT-based interventions. The low level of students’ perception of basic psychological needs indicates that students have less experience of need support interventions. First, students’ perception of autonomy needs depends on the autonomous learning environment and opportunities. Online teaching creates an autonomous environment for students, allowing students to choose learning content, time, path, and participate in learning according to their own way, improving students’ perception of autonomy needs (Holzer et al., 2021). However, students have to participate in online learning at home according to the school schedule during the epidemic in China. Teachers still provide students with control support. The control support may weaken students’ perception of autonomy needs (Ryan and Deci, 2020). Second, most K-12 students don’t have online learning experience before the epidemic, resulting in a low perception of their ability to be competent in online learning (Bai and Gu, 2021). Moreover, the separation between teachers and students results in students not getting teacher help immediately, the large number of tasks in online learning, and the lack of teacher guidance and feedback may weaken students’ perception of competence needs (Hartnett, 2015). Relatedness requires students to interact actively with others and provide mutual support (Hodges et al., 2020). However, during the epidemic, students were learning independently and lacked opportunities to interact with their teachers and peers, which directly weakened students’ perception of relatedness.

Students’ perception level of competence needs (23.16) is the highest, followed by relatedness (15.69); autonomy needs (12.17) is the lowest. This finding is inconsistent with Huang and Wang (2023), which found that college students’ perception of autonomy needs is the highest, followed by competence needs; relatedness is the lowest. One possible explanation is that, with fewer compulsory courses, college students have a lot of time and opportunities to choose other learning content, path, and time, thus having a high perception of autonomy needs. Unlike college students, most of the time, K-12 students’ learning activities follow the school schedule rather than being determined by themselves. Our finding is consistent with the argument that emergency online learning completely conflicts with students’ autonomy needs and self-determination (Hodges et al., 2020). Chiu (2022) claims that online environments are less supervised, giving students more autonomy to choose preferred learning materials and strategies and manage their learning. However, whether online learning can ultimately promote students’ perception of basic psychological needs depends on how it is delivered. Online learning can be provided in either controlling or autonomy-supportive ways. Moreover, meeting students’ autonomy needs is difficult given assessment and teachers’ traditional understanding of students’ roles (Wong, 2022). This tension highlights a critical gap between the theoretical affordances of digital learning and its actual implementation in highly regulated educational systems. Furthermore, one possible explanation is the argument forwarded by some cross-cultural researchers that autonomy needs do not apply to the Eastern collectivistic culture (Markus et al., 1996). In collectivist contexts, autonomy may manifest as “relational autonomy,” which refers to the freedom to act in alignment with family or group expectations, rather than as individual choice *per se*. Our instrument, likely developed in Western individualistic settings, may not fully capture this culturally embedded form of autonomy, potentially underestimating its presence among Chinese students.

### 5.2 Discussion for RQ2: group differences

The present study found significant group differences in students’ perception of basic psychological needs in different grades. Overall, middle school students’ perception of basic psychological needs satisfaction was significantly higher than that of elementary and high school students, and elementary school students were significantly higher than high school students. This finding supports the argument that the same intervention (online teaching) can be need satisfying for one group (middle and elementary school students) but not for another (high school students) (Ryan and Deci, 2002) Even though students all experience the same online education, their perception of basic psychological needs significantly differs because of individual differences. Among individual differences, students’ educational level affects their perception of the three basic psychological needs, and students with different educational levels have different perception levels of basic psychological needs (Chiu, 2022). Our study further answers how students at different levels of education affect their perception of basic psychological needs. Compared with middle school students, elementary school students have a low sense of autonomy, their ability is still in the early stage of development, and the ability to establish contact with others is still weak, which leads to their relatively lower perception level of autonomy and competence needs, as well as relatedness. Middle school students are in adolescence, and their autonomy consciousness is strong. Adolescence is the key period for forming self-confidence and possessing a strong sense of self-efficacy. Furthermore, they begin to experience various relationships with families, teachers, and others and are good at forming connections with others. Therefore, middle school students have the highest perception level of the three basic psychological needs. High school students need to learn more courses, understand and master increasingly profound knowledge, and face great pressure to continue their studies, which has a negative influence on the perception of basic psychological needs (Hartnett, 2015), resulting in the lowest perception level of the three basic psychological needs. This developmental trajectory aligns with Eriksoneeds. This developmental Erikson, 1968), which posits that early adolescence (typically corresponding to middle school) is characterized by identity exploration and social bonding, whereas late adolescence (often encompassing high school) is marked by role confusion and intense performance pressure. These challenges may suppress basic psychological need satisfaction in high-stakes academic environments. This study represents the first attempt to examine students in different grades’ perceptions of the three needs in online learning. Therefore, future research is needed.

The present study found no significant group differences in the perception level of basic psychological needs of students of different genders. Barrett and Lally (2010) noted significant differences in social and interactive behaviors. Female students were more social and interactive and expressed more interactive messages than males. Lowes et al. (2016) found female students more active than males. Moreover, Rovai and Baker (2005) indicated that students of different genders have different online learning experiences; female students believe online learning is more beneficial and absorb more knowledge in online courses. Thus, we expect that females might at least have significantly higher perception levels of relatedness and competence needs than males. Unfortunately, our hypothesis was not supported in the present study. However, the findings are consistent with related studies focusing on gender differences in online learning. Existing studies have found no significant differences between genders in online learning outcomes (Yu and Yu, 2021), students’ engagement, and performance (Krasodomska and Godawska, 2020). Recently, Turk et al. (2022) revealed that students’ perception of teaching and social and cognitive presence positively influence their perception of basic psychological needs satisfaction. One of our previous studies found no gender differences in students’ perceptions of teaching, social, and cognitive presence (Bai and Gu, 2021). Therefore, no gender differences are found in the perception of basic psychological needs. However, it should be noted that the absence of gender differences in self-reported psychological needs does not necessarily imply identical motivational processes; future research could explore whether different pathways lead to similar need satisfaction levels across genders.

Theoretically, basic psychological needs claim that individuals of different characteristics perceive the three basic psychological needs differently. Our research has further enriched this argument: an individual’s perception of basic psychological needs is influenced by grade (age) rather than gender.

#### 5.3 Discussion for RQ3: influence on engagement

The present study found that autonomy and competence needs significantly accounted for variance in students’ three online learning engagement dimensions. First, students’ perceptions of autonomy support in online learning give them more latitude to choose learning goals, resources, and strategies, which might encourage them to invest more willingness to participate in learning (emotional engagement) (Hew, 2016; Reeve et al., 2004). Emotional engagement is an important predictor of students’ cognitive and behavioral engagement (Connell and Wellborn, 1991). Therefore, a higher level of autonomy needs can bring a high level of behavioral and cognitive engagement. Second, when students perceive a higher level of competence needs, they will be effectively stimulated to participate in learning actively and then show obvious learning behaviors (behavioral engagement). Meanwhile, the perception of competence needs can help students to have a positive emotional response to learning (emotional engagement). Furthermore, when students have a higher perception level of competence needs, they are more inclined to engage in learning using deep learning strategies (cognitive engagement) (Huang and Wang, 2023). From a practical significance perspective, the magnitude of the beta coefficients offers meaningful interpretation. For instance, a β = 0.236 for competence needs in relation to emotional engagement represents a medium effect size (Cohen, 1988) and accounts for approximately 5.6% of unique variance in the outcome. While this may appear modest statistically, in real-world online learning contextscome. While this may appear modest statistically, in realaningful interpretation. Forit reflects a practically meaningful influence. Similarly, the adjusted R^2^ values (ranging from 0.302 to 0.540) indicate that the model explains between 30 and 54% of the variance in engagement types. This is a substantial explanatory power in educational research, particularly in complex, self-regulated environments, suggesting that supporting basic needs can yield tangible improvements in student engagement.

This study finds that competence needs have the greatest influence on online learning emotional, behavioral, and cognitive engagement. This finding suggests that perceived competence plays a particularly salient role in its association with emotional, behavioral, and cognitive engagement in online K-12 learning contexts. The prominence of competence as a key correlate may be partially understood in relation to the cultural and instructional context. In Chinese educational settings, where teacher authority and collective harmony are emphasized, opportunities for student autonomy in online learning may be limited, as choices regarding content, pace, and interaction are often structured by instructors (Guo and Wan, 2022). Similarly, while relatedness is valued, it tends to manifest through emotional support and normative compliance rather than through cognitively activating peer collaboration. Within such an environment, motivation may be less driven by self-direction or peer-driven belonging and more oriented toward demonstrating academic masteryy be less driven by self less driven by selfh cognitively actice is closely tied to future opportunities and familial expectations in China, success in learning may carry strong social and emotional significance. This cultural emphasis on mastery and achievement could amplify the motivational weight of competence, potentially explaining its relatively stronger association with engagement outcomes in the present study. Notably, this pattern contrasts with Western SDT applications where autonomy often emerges as the strongest correlate (e.g., Ryan and Deci, 2020), underscoring the need for culturally situated interpretations of basic psychological needs. Finally, it is worth noting that, while grounded in the Chinese Kvement could amplify the motivational weight of competence, potentially explaining its relatively strongidualistic cultures—such as those in North America or Western Europeal needs.tonomy and independent inquiry are central to pedagogical values (Ryan and Deci, 2020), autonomy may play a stronger role in promoting cognitive engagement. In contrast, competence might carry less motivational weight where self-worth is less tied to academic performance. However, it is important to acknowledge the high intercorrelations among the predictors. As shown in Table 4, autonomy and competence are strongly correlated (*r* = 0.801, *r*^2^ = 64.2%), and competence and relatedness (*r* = 0.809, *r*^2^ = 65.4%) also share substantial variance. This suggests that the unique contributions of each need—particularly in regression models—should be interpreted with caution. While multicollinearity diagnostics (VIF < 4) indicate that it does not severely bias estimates, the high shared variance implies that these constructs may function synergistically in practice, and their effects are not entirely independent. Future studies could employ relative weight analysis to better disentangle their unique and joint contributions.

Unexpectedly, the present study found that relatedness did not significantly associated with online learning cognitive engagement. One plausible explanation lies in the distinct nature of cognitive engagement, which typically requires sustained attention, deep information processing, and independent effort. In online learning environments, particularly those that are asynchronous or characterized by low levels of interaction, strong social connections may in some cases divert attention from cognitively demanding tasks (Hsiao et al., 2023). Another explanation lies in the functional design of social interactions. Specifically, during the data collection period in the Chinese K–12 context, online instruction often relied on pre-recorded video lectures and teacher led live sessions via platforms such as DingTalk or Tencent Meeting (Li et al., 2023). Peer interaction tools were primarily used for attendance, announcements, or emotional support rather than for structured, cognitively activating collaboration. This pattern, which emphasizes administrative and emotional functions over cognitive collaboration, may explain the nonsignificant link between relatedness and cognitive engagement. Moreover, this emphasis reflects broader collectivist norms in Chinese education (Tan et al., 2021), where relatedness is expressed through group harmony, teacher student respect, and emotional belonging. Such norms support behavioral compliance and positive emotional experiences, thus enhancing behavioral and emotional engagement. However, cognitive engagement depends on independent thinking and self-regulated effort, which are not directly fostered by social cohesion alone. Furthermore, the design and use of online interaction tools may influence this relationship. Features such as Zoom breakout rooms or discussion forums often facilitate emotional bonding and collaborative participation, but they may not inherently support cognitively rigorous activities (Hrastinski, 2008). If these tools are primarily used for social interaction rather than for structured, cognitively activating collaboration—such as peer problem-solving, critical debate, or joint knowledge construction (Mercer and Littleton, 2007)—their contribution to relatedness may remain functionally distinct from, or even interfere with, cognitive engagement. An alternative interpretation is that the measure of relatedness in this study captured general emotional or social bonding rather than collaborative academic interactions. While relatedness reflects the sense of being cared for and connected to others, it does not necessarily entail intellectually engaging dialogue. Had we measured the quality or frequency of peer collaboration in cognitively demanding tasks (e.g., joint problem-solving, knowledge co-construction), a stronger association with cognitive engagement might have emerged. Finally, our findings contrast with Huang and Wang (2023), who found relatedness positively linked to behavioral and emotional engagement but negatively to cognitive engagement among university students in Hong Kong. This difference may stem from developmental and contextual factors, as older students engage in more autonomous and peer driven learning. Younger students, by contrast, may rely more on teacher support, fostering emotional investment without similar cognitive trade offs. However, further research is needed to validate this interpretation.

## 6 Conclusion and implications

First, regarding students’ perceived levels of basic psychological needs (RQ1), results show that most students in this sample report moderate satisfaction. Second, concerning group differences (RQ2), middle school students reported the highest need satisfaction, followed by elementary students, with high school students reporting the lowest. Third, regarding the influence of basic psychological needs on online learning engagement (RQ3), autonomy and competence needs significantly accounted for variance in students’ emotional, behavioral, and cognitive engagement in our sample, with competence accounting for the largest proportion of explained variance.

Theoretically, this extends SDT to the understudied context of Chinese online K–12 education. Practically, the results suggest that teachers in similar contexts can use technology to provide students with autonomy support, structured guidance, and relatedness support in online learning. Well-designed, SDT-informed interventions may have the potential to enhance students’ perceived satisfaction of basic psychological needs. However, teachers must consider the potential for poorly implemented online interventions to undermine students’ need satisfaction. Policy-wise, the lower need satisfaction observed among high school students in this study suggests that schools and districts operating under comparable conditions could consider wellbeing monitoring and targeted resource allocation for this group, such as through institutional mechanisms that promote equitable resource distribution in online education. Nonetheless, large-scale policy implications require validation through broader, longitudinal, or multi-regional studies.

Furthermore, the finding that students’ perceived levels of basic psychological needs significantly accounted for variance in online learning engagement in this context reinforces the central role of competence in driving engagement. Therefore, educators and instructional designers working in similar online Kis context reinforcea may consider prioritizing strategies to enhance students’ competence satisfaction to foster greater online learning engagement. For example, instructional design in such environments can incorporate timely, mastery-focused feedback enabled by learning analytics. In addition, personalized learning systems may offer promising avenues for supporting competence, though their effectiveness requires further empirical investigation.

### 6.1 Limitations and recommendations for future research

This study has several limitations. The sample was drawn exclusively from China, which may affect the generalizability of this study. Further studies should invite K-12 students from different cultures to reveal students’ perception of basic psychological needs and its influence. Moreover, the cross-sectional design limits causal interpretation, and the exclusive use of self-report data may introduce response bias. Future studies should employ longitudinal designs and triangulate data with behavioral or observational measures. Finally, this study only used correlation and regression analysis to examine the influence of basic psychological needs on online learning engagement. Future research should apply methods such as structural equation modeling to investigate the underlying mechanisms and test for moderating effects of variables like gender, grade level, and engagement type.

## Data Availability

The raw data supporting the conclusions of this article will be made available by the authors, without undue reservation.
